# Corneal transplantation: Restoring sight to the blind

**DOI:** 10.1111/aos.70155

**Published:** 2026-05-12

**Authors:** Claus Cursiefen

**Affiliations:** ^1^ Department of Ophthalmology University Hospital, University of Cologne Cologne Germany

## Abstract

Corneal blindness is one of the leading causes of vision loss worldwide. Transplantation of the cornea can restore vision and make blind patients see again. Corneal transplant surgery has undergone major evolution in recent decades with the advent of minimally invasive lamellar surgical approaches such as DMEK and DALK. Nonetheless, on a global scale, penetrating keratoplasty, especially in so‐called high‐risk settings, remains a commonly performed surgery. As pathologic blood and clinically invisible lymphatic vessels have been identified as key risk factors for immune reactions after high‐risk transplantation, we present current evidence for temporary pre‐transplant lymphangioregression in the cornea as a novel concept to promote graft survival after subsequent transplantation. Here, UVA‐based crosslinking can be used as a novel method to regress pathologic corneal vessels. Also, performing DMEK in high‐risk eyes and the use of artificial endothelial transplants can prevent immune reactions in these patients. Finally, novel developments in the area of DMEK, DSO and endothelial cell transplantation are discussed. We are experiencing (r)evolutionary improvements not only in modern lamellar but also in penetrating corneal transplantation to make patients with corneal blindness see again.

Dear President, Dear Ladies and Gentlemen, Dear Colleagues,

First of all, thank you very much for the great honour to deliver the 2025 Bjerrum Medal Lecture of the Danish Ophthalmological Society (DOS) here at the Rigshospitalet in Copenhagen.

This award, which has been presented since 1965 by the DOS, was given to many innovative, distinguished and dedicated colleagues in our field. Mr. President, in your introduction, you have already acknowledged the history of the medal and the significance of Prof. Dr. Bjerrum. In the field of corneal surgery, I would like to highlight four former Bjerrum Medalists whose work forms the basis of this lecture and our research activities: Prof. Isaac Michaelson from Jerusalem (award winner in 1967), who was the first to postulate a diffusible factor X that mediates corneal neovascularization and was later identified as VEGF‐A (Campbell & Michaelson, 1949; Maurice et al., 1966), Prof. Claes Dohlman from Boston, the “inventor” of the Boston keratoprosthesis (award winner in 1971; Dohlman & Doane, 1994), Prof. Gottfried O.H. Naumann from Erlangen (1995 award winner), who established non‐mechanical laser‐assisted corneal transplantation (Lang et al., 1989), and Prof. Jesper Hjortal from Aarhus (2019 award winner) for his pioneering work in the field of corneal crosslinking (Gustafsson et al., 2026; Søndergaard et al., 2013) on which the lecture is also based.

The “fight against blindness” is the original task that we as ophthalmologists face. Vision is the most important sensory function in humans, with more than a third of the human brain involved in processing sensory information. I am quoting here my mentor Prof. “Fritz” Naumann from Erlangen, who gave the Bjerrum Lecture in 1995. When people are asked what they fear most in terms of health, the fear of going blind always ranks first or second, similar to the fear of developing cancer. The idea of restoring sight to the blind is a familiar theme in literature and in many religions, as can be seen, for example, on the door of the Romanesque church of Maria im Kapitol in Cologne (Figure 1). Religious motifs always depict healing of the blind as an activity that takes place on the “surface of the eye.” That is—amongst others—why this lecture deals with corneal transplantation, which actually allows us to restore sight to the blind.

**FIGURE 1:**
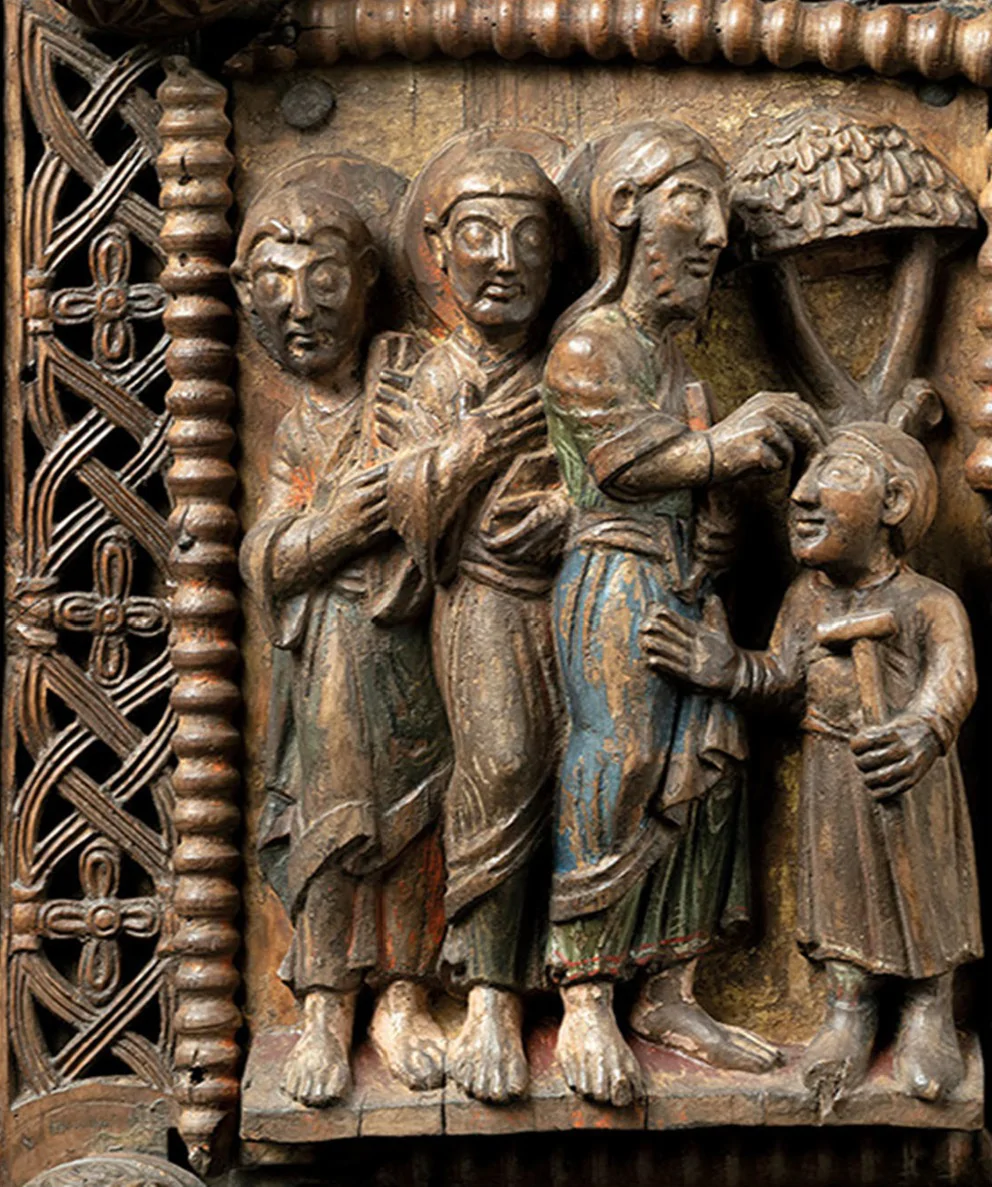
“Curing blindness.” Part of romanesque door relief at “St. Maria im Kapitol” from the 11th century, Cologne (*Source*: https://museenkoeln.de/portal/bild‐der‐woche.aspx?bdw=2016_51).

Figure 2 shows patients having undergone multiple surgical procedures on their last eye due to chemical burns, with hand movement before and visual acuity of up to 0.8 after Boston keratoprosthesis (Enders et al., 2019). This means that with a Boston keratoprosthesis, we can actually restore sight to blind people in their last remaining eye. What does not yet work, at least not functionally, is the transplantation of the entire eye. In 2024, such a transplant was performed with anatomical success, but without functional improvement.

**FIGURE 2:**
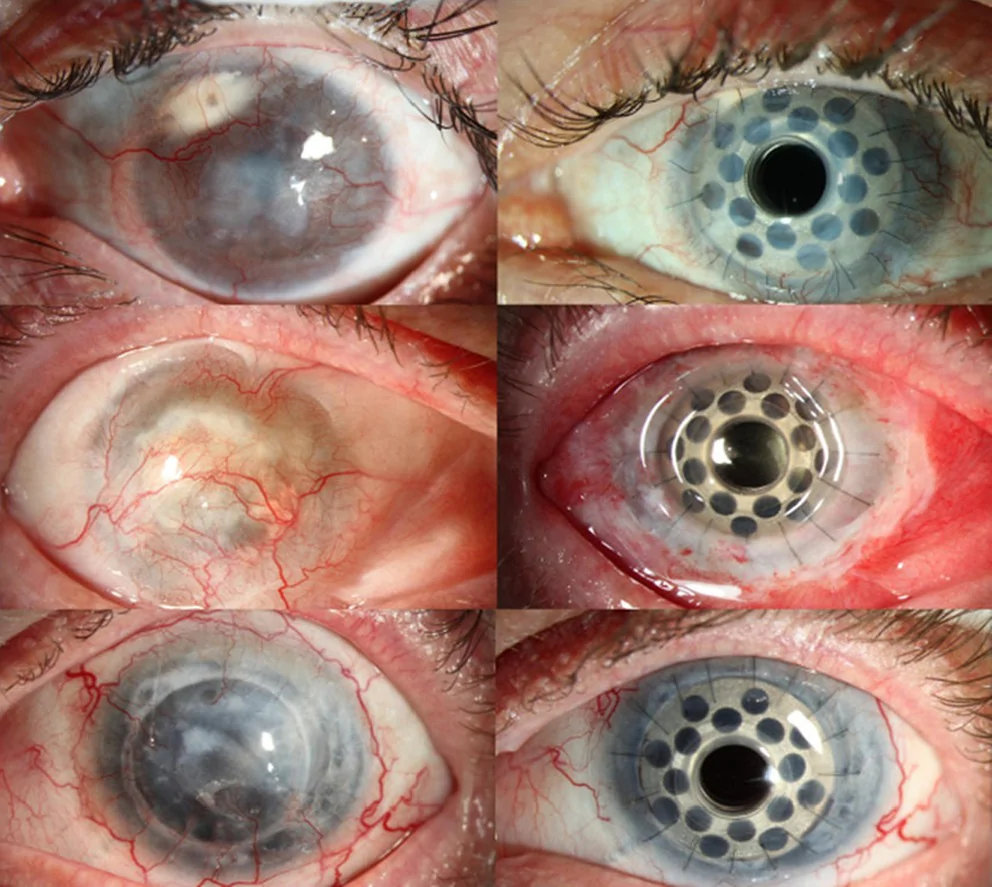
Boston Keratoprosthesis (Kpro) to treat vascularized scars after chemical burn and multiple failed previous surgeries (left side prior to Boston Kpro; right side: After surgery). Vision increased from hand movement up to 0.8 (*Source*: Schaub et al., 2021).

The lecture is divided into four parts: (i) the importance of corneal lymphatic vessels and UVA cross‐linking for improving corneal transplant survival, (ii) the topic of corneal donor shortage, (iii) an update on high‐risk keratoplasty and finally (iv) an update on new developments in DMEK and more modern procedures for the treatment of Fuchs' endothelial corneal dystrophy.

## THE IMPORTANCE OF CORNEAL LYMPHATIC VESSELS AND UVA CROSSLINKING FOR IMPROVING CORNEAL HIGH‐RISK TRANSPLANT SURVIVAL

On the first topic: The field of corneal transplant surgery has changed dramatically in recent years. Data from the German Ophthalmological Society's (DOG) transplant registry show that over the last 10 years, the proportion of minimally invasive lamellar posterior keratoplasties has increased from around 20% to around 70% (Flockerzi et al., 2018, 2024). This is because DMEK treatment of Fuchs' endothelial dystrophy delivers very good visual acuity results in a very short time and is associated with a very low risk of rejection (2.3% in 5 years) (Hos et al., 2019; Hos, Tuac, et al., 2017; Hos, Bukowiecki, et al., 2017). Globally (and especially in lower and middle‐income countries [LMIC]), however, the majority of patients is still treated with penetrating transplants (Matthaei et al., 2017). The vast majority of these also take place in vascularized, so‐called high‐risk situations, e.g., 75% of transplants in India. There is therefore a global unmet need to improve graft survival, especially in vascularized high‐risk eyes, and to prevent graft rejection.

The normal cornea is physiologically avascular. The so‐called corneal “lymph” angiogenic privilege ensures the abrupt transition from vascular conjunctiva to avascular cornea. Multiple mechanisms ensure this evolutionarily highly conserved phenomenon (Cursiefen, 2007; Cursiefen, Cao, et al., 2004; Cursiefen, Chen, et al., 2004; Cursiefen, Masli, et al., 2004) Figure 3. Of particular interest is the ectopic expression of VEGF receptors in the epithelium of the avascular cornea. These act as decoy receptors to bind and neutralize VEGF growth factors (VEGF A, C, D) and normally prevent vascular sprouting into the cornea (Cursiefen, Chen, et al., 2006; Cursiefen, Maruyama, et al., 2006; Ambati et al., 2006). With the exception of the manatee, all mammals therefore have a physiologically avascular cornea. Manatees have a mutation in the VEGFR3 gene (Ambati et al., 2006). Pathological vascularization of the cornea not only reduces vision per se, but also increases the chance of a rejection reaction after a subsequent corneal transplant. This risk of rejection increases with the number of corneal quadrants affected (Bachmann et al., 2010).

**FIGURE 3:**
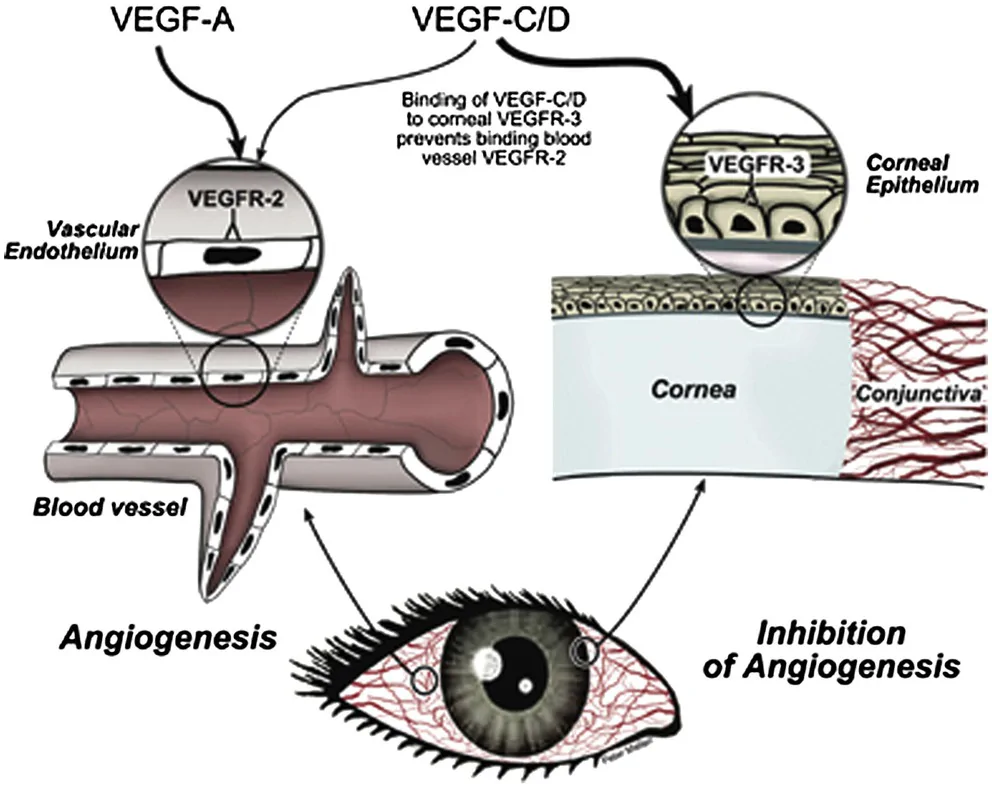
Corneal (lymph)angiogenic privilege is evolutionarily highly conserved and maintained by numerous mechanisms. One of these is the ectopic expression of VEGF receptor 3 on corneal epithelial cells. These receptors act as a “cytokine sink,” neutralizing angiogenic growth factors and thereby normally maintaining avascularity (*Source*: Peter Mallen, Schepens Eye Research Institute, Boston).

As early as 1948, Isaac Michaelson (Bjerrum medalist 1967) postulated the existence of a diffusible and soluble growth factor X, which is emitted from the site of inflammation and attracts vessels (Campbell & Michaelson, 1949). This factor was later identified as VEGF‐A/VPF. We can also observe this in everyday practice, where it has been shown that after penetrating keratoplasty with double running Hoffmann sutures, in over 90% of cases vessels grow from the limbus to the outer turning point of the corneal sutures (as the site of release of angiogenic growth factors) and then grow centrally along the suture material (Cursiefen et al., 2001; Cursiefen, Schlötzer‐Schrehardt, et al., 2002; Cursiefen, Martus, et al., 2002) Figure 4. Vascular growth is therefore most prominent in the 6 and 12 o'clock areas, where the distance to the limbus is shortest (Cursiefen et al., 2001; Cursiefen, Martus, et al., 2002; Cursiefen, Schlötzer‐Schrehardt, et al., 2002).

**FIGURE 4:**
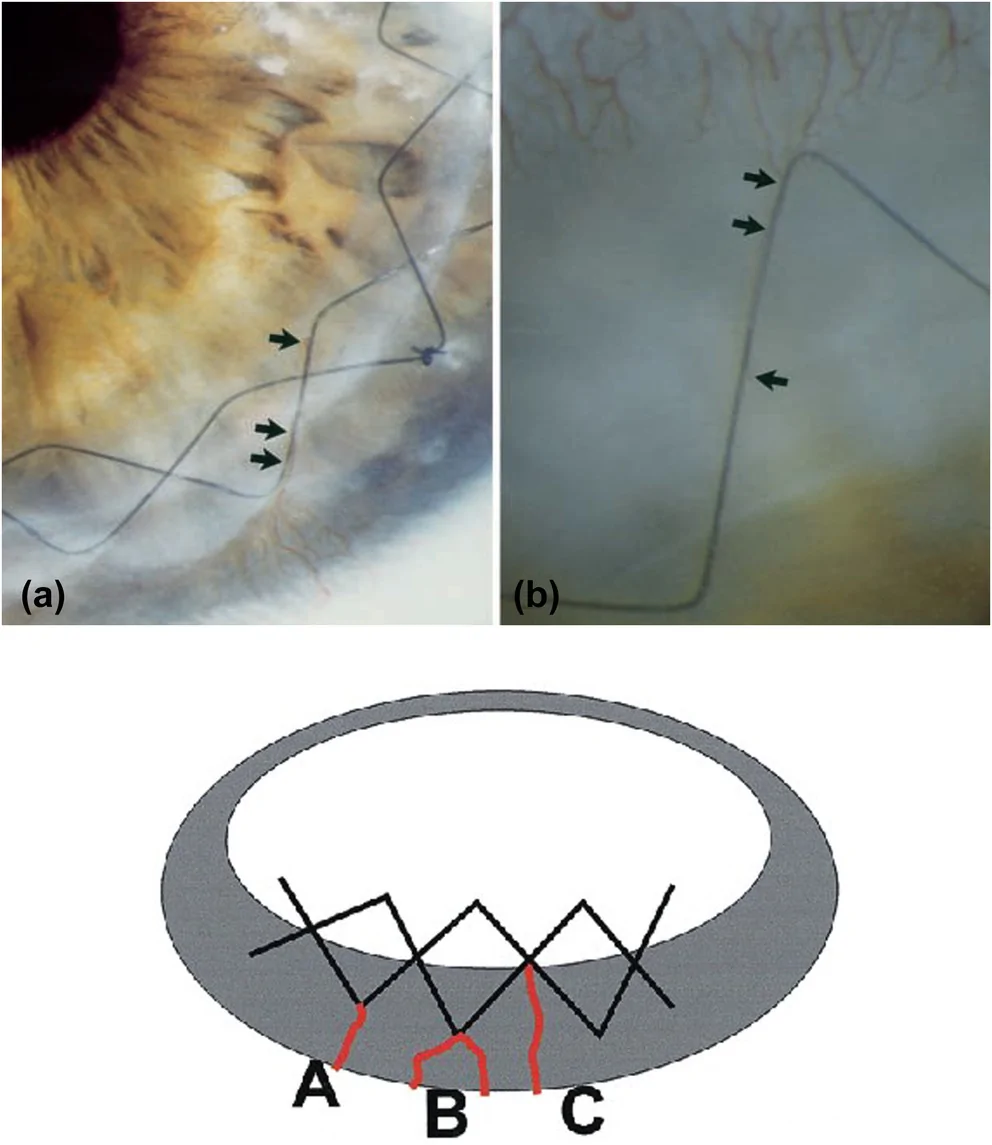
Corneal neovascularization after normal‐risk keratoplasty is usually directed toward the outer turning points of the double continuous sutures (a). This is where the diffusion distance of angiogenic factors to the limbus is shortest. Afterwards, vessels follow the suture track (b, c). (*Source*: Cursiefen et al., 2001).

Now to the topic of lymphatic vessels and their importance for graft survival. Are there lymphatic vessels in the cornea? These are clinically invisible and therefore their existence was unclear for a long time. In the early 2000s, using new specific markers for lymphatic vascular endothelium such as LYVE‐1, VEGFR3 and Podoplanin (Cursiefen, Chen, et al., 2003, 2006; Cursiefen, Hofmann‐Rummelt, et al., 2003; Cursiefen, Martus, et al., 2002; Cursiefen, Maruyama, et al., 2006; Cursiefen, Schlötzer‐Schrehardt, et al., 2002), we were able to clearly show for the first time that, in addition to clinically visible blood vessels, there are also clinically invisible lymphatic vessels in human corneas (Figure 5; Cursiefen, Martus, et al., 2002; Cursiefen, Schlötzer‐Schrehardt, et al., 2002). This applies to both vascularized murine (Cursiefen, Cao, et al., 2004; Cursiefen, Chen, et al., 2004; Cursiefen, Masli, et al., 2004) and human corneas (Cursiefen, Schlötzer‐Schrehardt, et al., 2002; Cursiefen, Martus, et al., 2002). The late identification of lymphatic vessels in the normal cornea was due to the decades‐long lack of specific markers for lymphatic vascular endothelium (Schroedl et al., 2014). Aselius described the presence of a second vascular system as early as 1627, but it was not until 1995 that Alitalo and colleagues from Helsinki succeeded in identifying the first specific marker, VEGF receptor 3 (Kaipainen et al., 1995).

**FIGURE 5:**
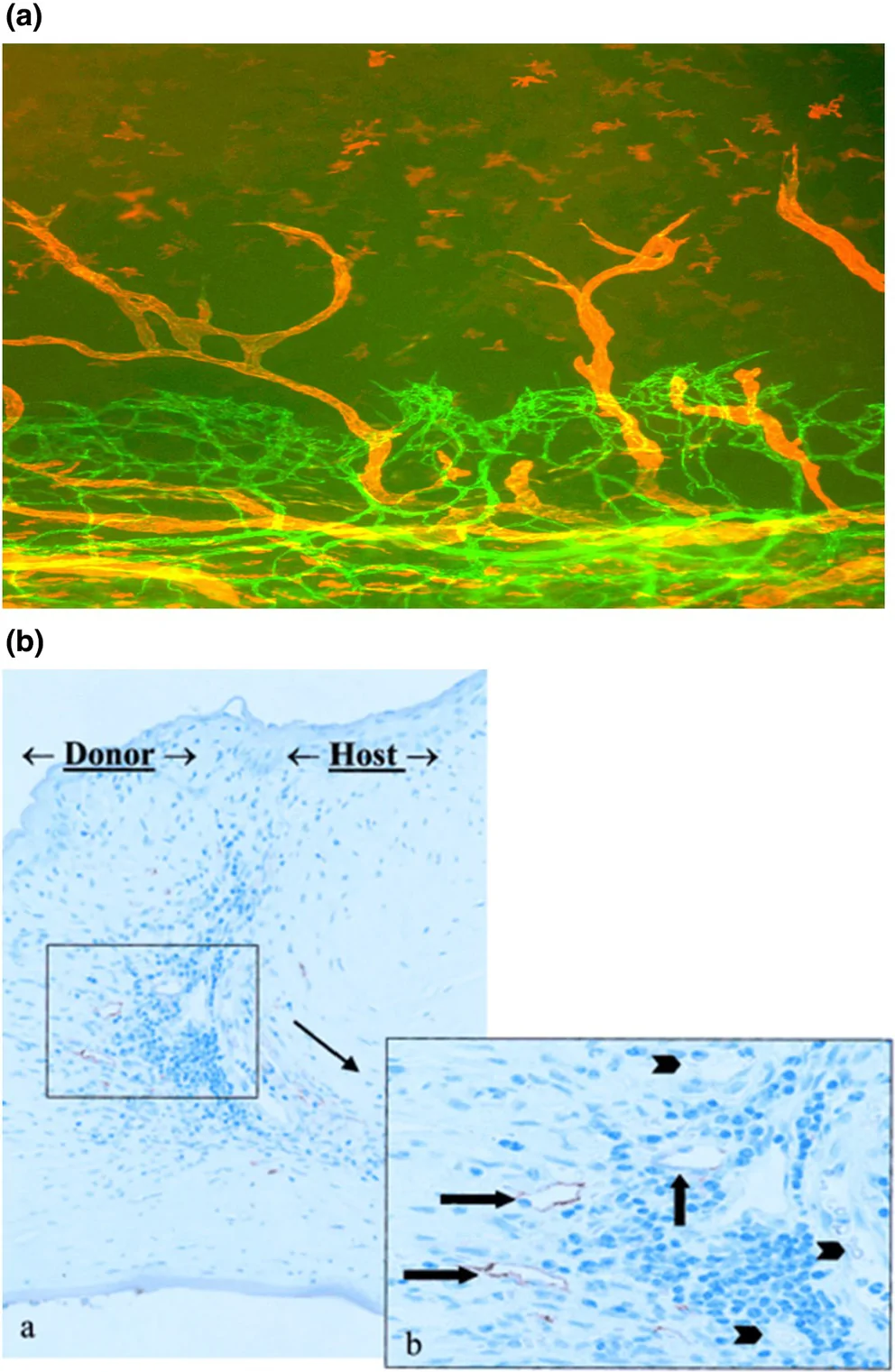
Clinically invisible lymph vessels in vascularized corneas can be visualized using specific markers (such as Podoplanin, LYVE‐1 and VEGFR3). Here in (a) a pathologically vascularized murine cornea displays blood vessels in green (CD31) and lymphatic vessels in red (LYVE‐1) at the limbal arcade growing into the cornea. Note LYVE‐1 positive macrophages in the cornea. *Source*: Cursiefen, Chen, et al., 2004 . (b) Podoplanin‐stained lymphatic vessels in a vascularized human cornea. Note inflammatory cells around the vessels (B from: Cursiefen, Schlötzer‐Schrehardt, et al., 2002).

Building on this, we and other groups were then able to show in various experiments that, in a high‐risk context, the lymphatic vessels (at least in the mouse model) are the more decisive player in the induction of immune reactions after corneal transplantation (Dietrich et al., 2010). These lymph vessels can also be detected in vascularized inflamed human corneas particularly early after an inflammatory stimulus and then regress over time (Cursiefen, Chen, et al., 2006; Cursiefen, Martus, et al., 2002; Cursiefen, Maruyama, et al., 2006; Cursiefen, Schlötzer‐Schrehardt, et al., 2002). This means that in vascularized high‐risk eyes, both arms of the so‐called immune reflex arc (afferent lymph vessels and efferent blood vessels) are present (Figure 6). This explains the dramatic difference in graft survival in immunoprivileged avascular normal‐risk eyes versus pathologically vascularized high‐risk eyes.

**FIGURE 6:**
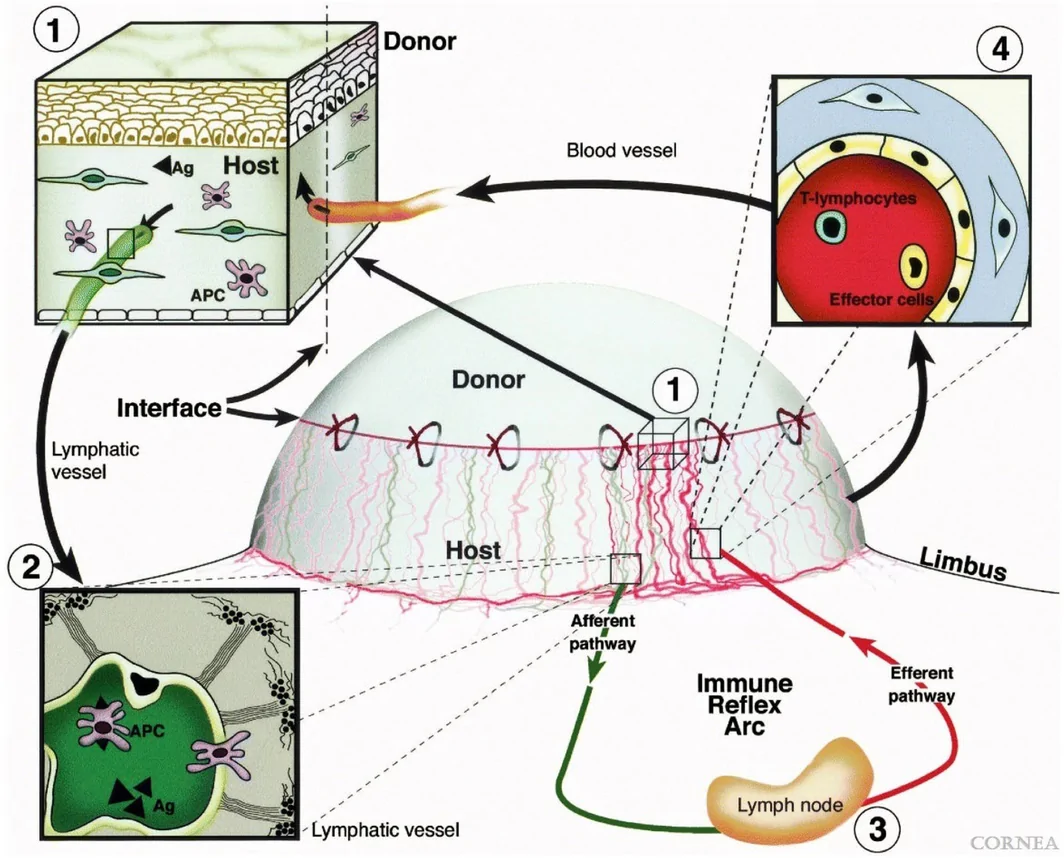
Immunoreflex arc of the immune response after high‐risk keratoplasty mediated by afferent lymph vessels and efferent blood vessels in vascularized eyes (*Source*: Cursiefen, Chen, et al., 2003).

What are the therapeutic options against these blood and lymph vessels in high‐risk eyes to promote transplant survival? There is extensive preclinical data showing that inhibiting pathological blood and lymph vessel sprouting into the cornea prior to transplantation improves subsequent graft survival after high‐risk transplantation (Hou et al., 2021). In addition, we and others have shown that inhibiting additional, surgery‐induced vascular sprouting also improves graft survival (Cursiefen, Cao, et al., 2004; Cursiefen, Chen, et al., 2004; Cursiefen, Masli, et al., 2004). In routine clinical practice and globally, however, the bigger problem is that patients already have pre‐existing, mature pathological blood and lymph vessels that generally respond poorly to topical angiogenesis inhibitors (Cursiefen, Chen, et al., 2003; Cursiefen, Hofmann‐Rummelt, et al., 2003).

There are various “physical” ways to destroy these vessels, e.g., fine‐needle cauterization (Le et al., 2018) or PDT (Hou et al., 2017). In addition, we recently identified UVA‐mediated corneal crosslinking as a gentle method of destroying pathological corneal blood and lymph vessels in animal models (Hou et al., 2018). This method was then used in subsequent transplants in cross‐linked murine high‐risk corneas, resulting in a significantly better graft survival rate (Figure 7; Hou et al., 2018). UV crosslinking can also be used to regress pathological blood vessels in patients. In a pilot study involving five patients, Schaub et al. were able to show that this leads to a regression of vessels (Schaub, Hou, et al. 2021). Price and colleagues recently demonstrated in 38 patients that crosslinking is particularly effective in cases of inflammatory and transplant‐associated vascular sprouting (Price Jr et al., 2025). However, if the vascular stimulus persists, UVA irradiation is not as effective or must be repeated. CXL with higher fluence appears to be more effective in regressing corneal blood vessels compared to the standard UVA fluence used in corneal crosslinking (Donner et al., 2024).

**FIGURE 7:**
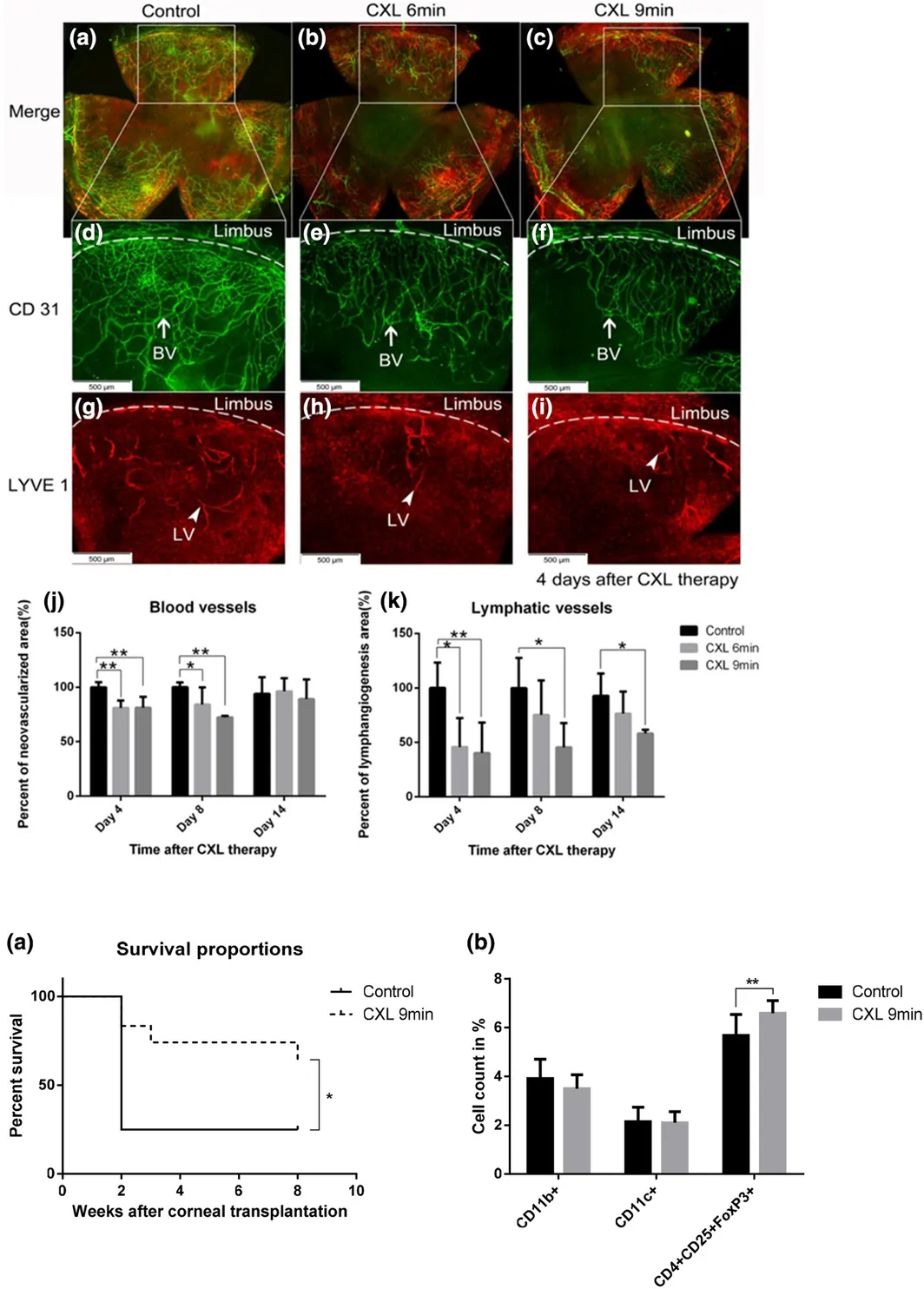
UVA‐mediated corneal crosslinking destroys pathological corneal blood and lymph vessels and improves graft survival after subsequent high‐risk (murine) corneal transplantation (*Source*: Hou et al., 2018). CD31 stains blood and LYVE‐1 lymphatic vessels.

Clinical studies are needed to bring the new concept of temporal, pre‐transplant lymphangioregression to improve graft survival in vascularized high‐risk eyes into clinical practice. The CrossCornealVision study (EudraCT number 2015‐002726; Figure 8; Wiedemann, Hos, Limburg, et al., 2024; Wiedemann, Hos, Mestanoglu, et al., 2024) has been running in Germany as a multicentre, prospective, randomized study since 2023. At eight centres, patients with high‐risk eyes are either treated once or twice with UVA crosslinking and then transplanted, or transplanted only. In the treatment group, remaining vessels can also be destroyed by vascular cauterization at the time of surgery. The study is expected to include a total of 110 patients; currently, more than 50% have been recruited, and results are expected in 2027 (Wiedemann, Hos, Limburg, et al., 2024; Wiedemann, Hos, Mestanoglu, et al., 2024).

**FIGURE 8:**
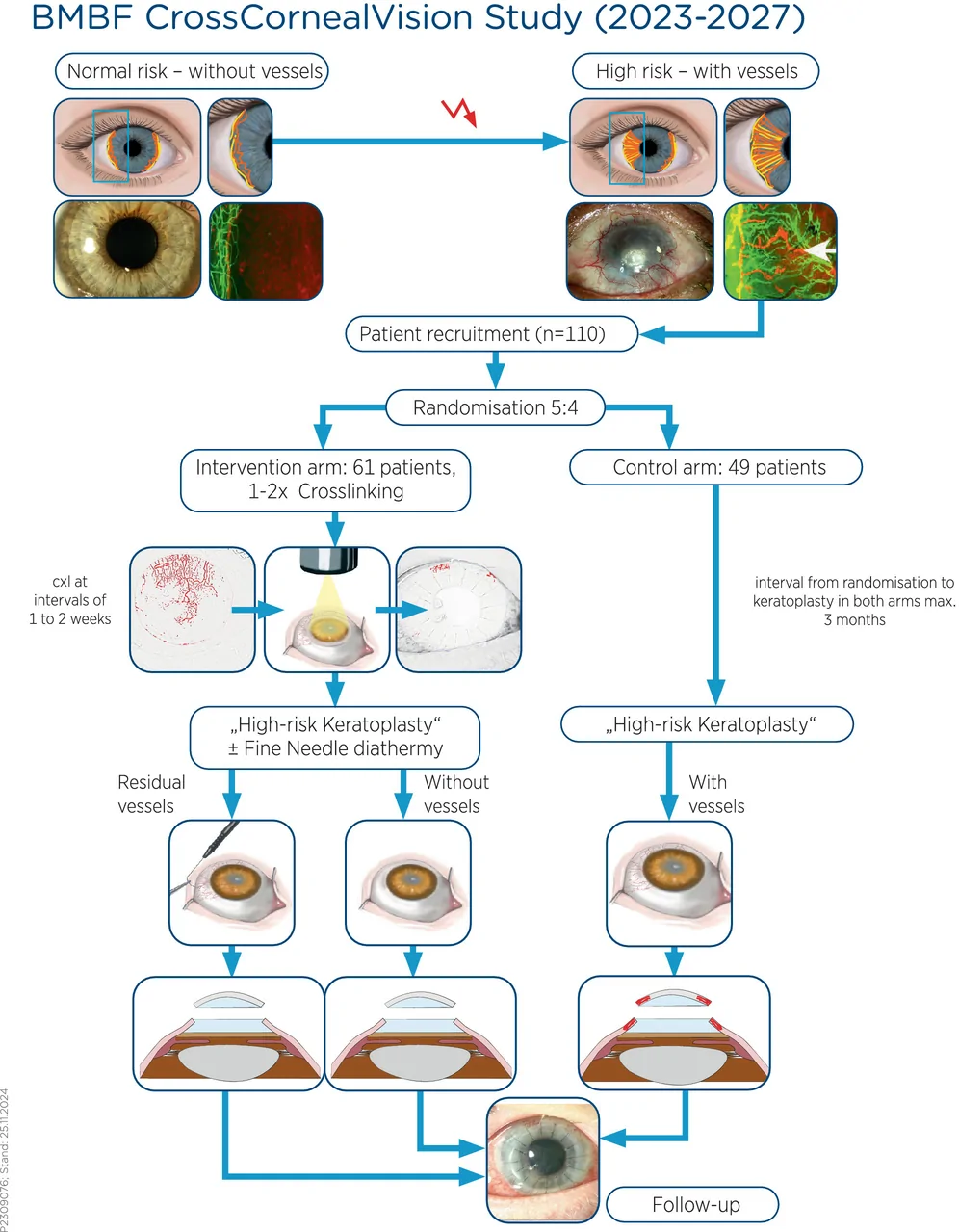
Study design of the CrossCornealVision study: Graft survival is compared between high‐risk eyes with and without preoperative crosslinking treatment. This tests the new therapeutic concept of temporary pre‐transplant lymphangioregression through UVA irradiation to promote subsequent high‐risk corneal transplant survival (*Source*: Wiedemann, Hos, Limburg, et al., 2024).

Beyond this study approach, a mouse model of corneal transplantation has shown that the combination of preconditioning of donor and recipient (donor by Eylea incubation and recipient by corneal cross‐linking) shows dramatically improved graft survival compared to isolated pretreatments with cross‐linking or Eylea (W. Zhang, F. Bock, C. Cursiefen, Unpublished data). Building on this, we have experience with three pilot patients to date who, with multiple previous transplant rejections, received preconditioning of the donor using Eylea and of the recipient using cross‐linking. So far, this treatment has been well tolerated, with no immune reaction observed in any of the patients (at follow‐up of up to 1.5 years). This means that further studies are needed to analyze whether this single‐stage procedure involving combined preconditioning of both donor and recipient can also significantly improve transplant survival.

In addition to the negative effect of lymphatic vessels in the area of transplantation, lymphatic vessels also appear to have a positive effect in the cornea. For example, in cases of acute fluid influx and stromal oedema in acute keratoconus, the presence of isolated lymphatic vessels has been demonstrated in explanted human corneas Figure 9 (Hos, Bukowiecki, et al., 2017; Hos, Tuac, et al., 2017). This can also be replicated in mouse models. Interestingly, blocking oedema‐induced lymphangiogenesis via VEGF R3 leads to delayed resorption of stromal oedema (Hos, Tuac, et al., 2017; Hos, Bukowiecki, et al., 2017). This may therefore provide a potential drug therapy option for corneal oedema in the future.

**FIGURE 9:**
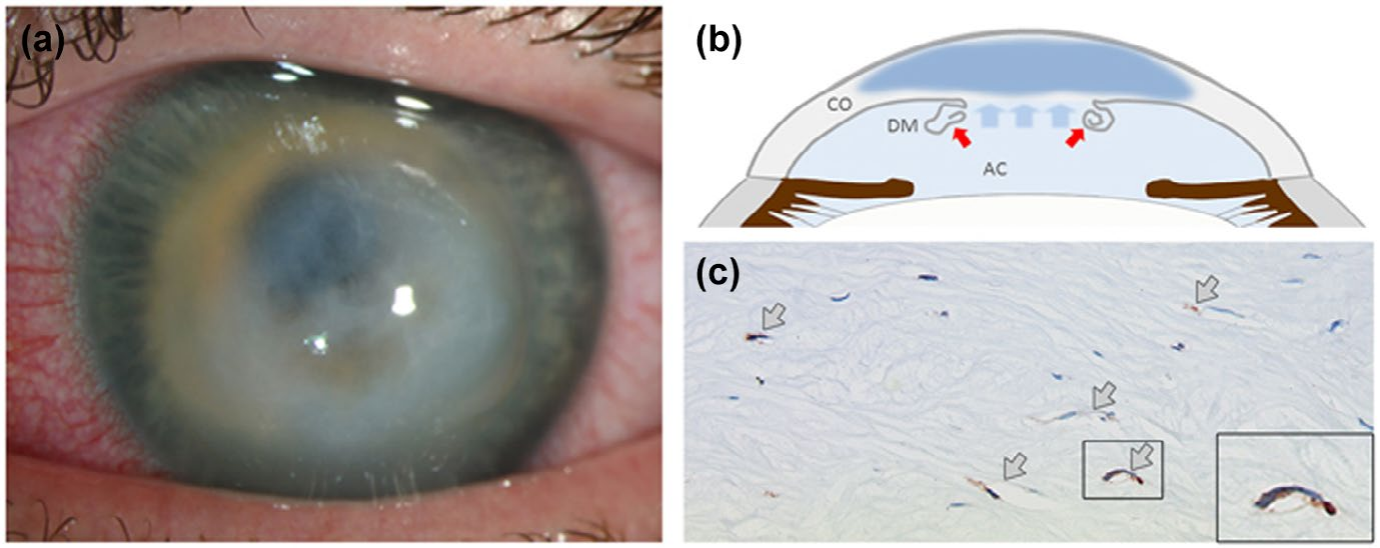
Isolated corneal lymphangiogenesis after acute hydrops in keratoconus. LYVE‐1 positive lymphatics seem to be involved in drainage of tissue fluid. *Source*: Hos, Bukowiecki, et al., (2017).

Current treatment options for acute keratoconus include suture and gas instillation (Yahia Chérif et al., 2015) and IOCT‐guided puncture of residual stromal fluid (“triple approach”; Bachmann et al., 2019) in acute keratoconus or mini‐DMEK (Bachmann et al., 2019). Building on the work of Jesper Hjortal (Bjerrum Lecture from 2019; Søndergaard et al., 2013; Ivarsen & Hjortdal, 2013), we were able to show in 2024 that in pre‐crosslinked corneas that develop acute keratoconus, the clinical manifestations of keratoconus are significantly reduced (Howaldt et al., 2024).

In summary, we currently have fascinating new options to temporarily regress pathologic corneal blood and lymphatics in high‐risk corneas. This novel concept of “temporary, pretransplant lymphangioregression in high‐risk eyes to promote graft survival” is currently being tested in prospective clinical trials. On the other hand, isolated corneal lymphangiogenesis may be a new tool to treat corneal oedema.

## CORNEAL TRANSPLANT DONOR SHORTAGE: WHAT CAN BE DONE?

Corneal transplantation is the most commonly performed form of tissue transplantation, with approximately 11 000 operations per year in Germany and approximately 1200 per year at the University Eye Clinic in Cologne (Flockerzi et al., 2024). This results in a significant shortage of donors. Although the willingness to donate organs and tissue in Germany is generally positive (80% in surveys), in the absence of an opt‐out solution, only 60% of those surveyed have given it serious thoughts and only 40% have a donor card. There is therefore a clear need for improvement in this area, which is why the German Ophthalmological Society (DOG) is attempting to raise awareness of the issue of organ and tissue donation with its “#SpendeamEnde” campaign (https://dog.org/hornhaut‐spendeamende).

Another option for saving corneal donor tissue that is already feasible is the split cornea transplantation concept. Here, the donor cornea is divided into two parts so that the front part can be used for a DALK and the back part for a DMEK (Heindl et al., 2011). A retrospective analysis of over 1000 donor corneas showed that the split cornea concept safely and effectively combats donor shortages, allowing one cornea to be used for two recipients (Schaub et al., 2015; Figure 10).

**FIGURE 10:**
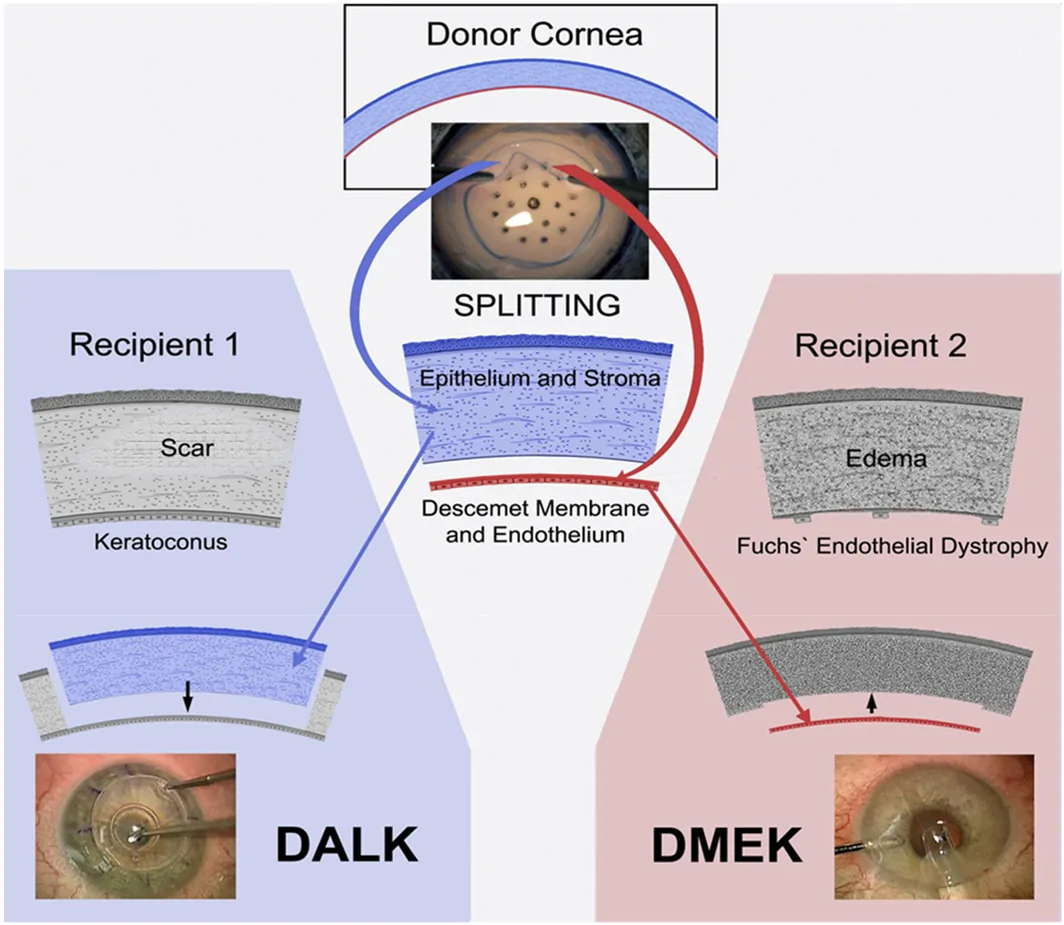
The split cornea concept allows ONE donor cornea to be used for TWO patients by combining DMEK and DALK surgery (*Source*: Heindl et al., 2011).

## WHAT'S NEW IN THE FIELD OF HIGH‐RISK TRANSPLANTATION?

First, an update on the topic of “high‐risk DMEK.” What does this mean? Recent studies show that DMEK can also be performed in vascularized eyes with reversible stromal oedema. This new approach was first described in a 36‐year‐old female patient after severe chemical burns (Hung Le et al., 2019). Unexpectedly, it was observed that not only did the stromal oedema of approximately 1200‐μm corneal thickness with pronounced stromal neovascularization and visual acuity of 20/400 regress to a corneal thickness of 580 after DMEK with iOCT, but the cornea also cleared up and the vessels regressed (with visual acuity increasing to 0.6; Hung Le et al., 2019; Figure 11). In a subsequent larger retrospective study by Hayashi and colleagues on 24 eyes from the Cologne DMEK database, it was shown that a significant improvement in visual acuity could also be achieved in the other vascularized high‐risk eyes with stromal oedema (Hayashi et al., 2021). This was achieved with only a mildly increased immune response risk of 4% and significant regression of blood vessels (Hayashi et al., 2021). In other words, the use of “high‐risk DMEK” in vascularized corneas with oedema but without scarring can avoid the high risk of rejection associated with alternative penetrating keratoplasty.

**FIGURE 11:**
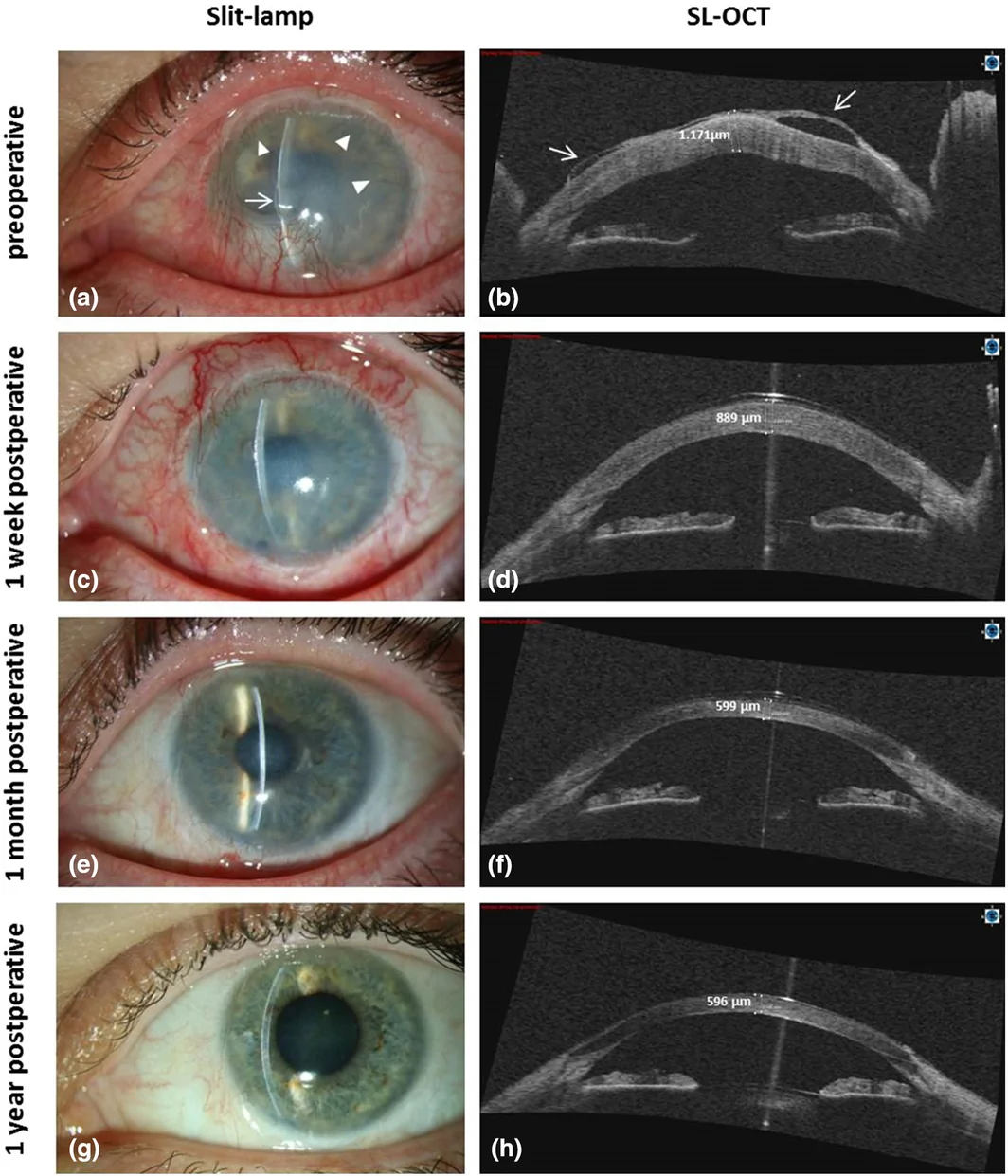
High‐risk DMEK in vascularized oedematous cornea leads to a reduction in stromal oedema, regression of blood vessels and improved visual acuity of 0.6 (*Source:* Hung Le et al., 2019). Patient suffered from chemical burn before.

Another alternative to high‐risk DMEK in vascularized oedematous corneas is now available in the form of EndoArt, an artificial inner layer which, however, only reduces swelling in the central cornea (Auffarth et al., 2021; Wiedemann, Hos, Mestanoglu, et al., 2024). This 6.5 mm plastic disc must be fixed in place with sutures and also pressed with gas (12% C3F8). In a study by Wiedemann and colleagues (Wiedemann, Hos, Mestanoglu, et al., 2024), we were able to show that EndoArt is a valid treatment option, especially in eyes with a high risk of repetitive DMEK failure, e.g., in glaucoma implants, and reduced visual acuity prognosis.

The maximum therapy in high‐risk eyes is the Boston keratoprosthesis established by Claes Dohlman (Bjerrum Lecture 1971; Dohlman & Doane, 1994), which can achieve significant visual improvement in the majority of patients. Unfortunately, there are numerous long‐term complications, so this is a temporary vision‐improving procedure. Telemetric pressure measurement can easily alleviate the problem of glaucoma‐associated complications with Boston Kpro (Enders et al., 2019).

## UPDATE: WHAT ARE THE LATEST ADVANCES IN THE TREATMENT OF FUCHS' ENDOTHELIAL DYSTROPHY?

For over 100 years, the entire cornea has been replaced in cases of endothelial corneal disease, even though this represents excessive overtreatment. For Fuchs' corneal endothelial dystrophy, which affects about 5% of the population over 50 years of age, DMEK, first performed by Gerrit Melles in 2006, is therefore the treatment of choice (Melles et al., 2006). This is currently the most frequently performed transplant at most transplant centres in Germany (Flockerzi, et al., 2024). What are the latest developments in this area?

### Immune rejection reaction

The risk of rejection in the normal‐risk DMEK range is 2.3% after 5 years (Hos, Tuac, et al., 2017). Most patients are asymptomatic during the immune reaction, which means that they should be checked regularly even if they have no symptoms. Most patients experiencing immune rejections were not on steroid therapy anymore, meaning they should be treated with eye drops once a day for at least 2 years, and the vast majority (except for 0.2%) can be cured with intensive topical steroid therapy (Hos, Bukowiecki, et al., 2017; Hos, Tuac, et al., 2017). This means that endothelial immune reactions after DMEK are less common, milder and more reversible than after penetrating keratoplasty (Figure 12).

**FIGURE 12:**
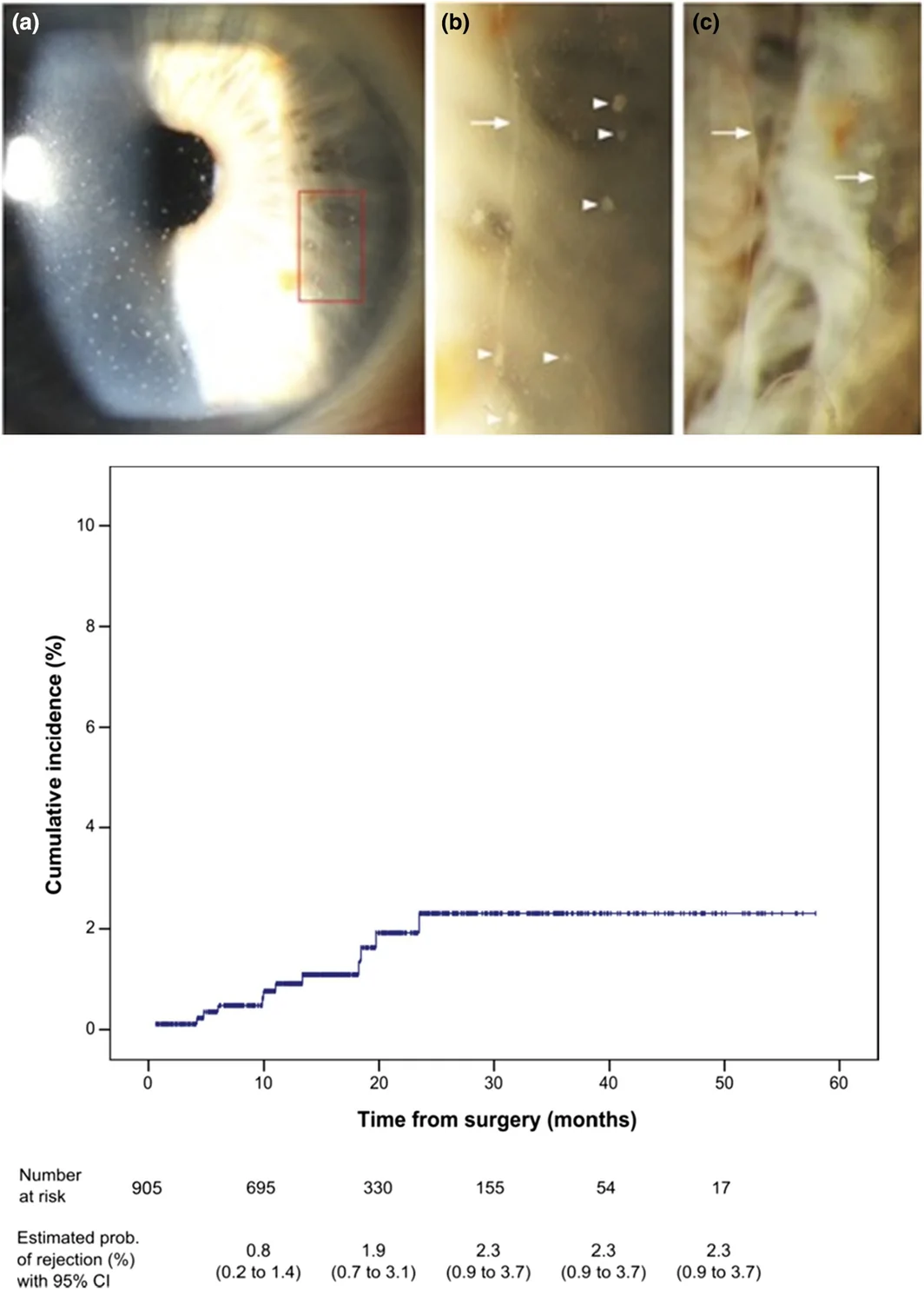
Immune reactions after DMEK are mild, often asymptomatic and cluster in the first 2 years with rejection rates around 2% (from Hos, Tuac, et al., 2017). (a) Overview of cornea after DMEK with mild immune reaction. (b) Note precipitates in an area of “nacked stroma” after DMEK having been covered by donor endothelial cells after transplantation. (c) After topical steroid therapy, precipitates disappear.

### Cystoid macular oedema after DMEK

Cystoid macular oedema was a common problem in the early years of DMEK and Triple DMEK (Figure 13). However, the risk can be reduced from about 13% to almost zero if intensive topical steroids (hourly instead of 5 times a day) are administered in the first week after surgery (Hoerster et al., 2016).

**FIGURE 13:**
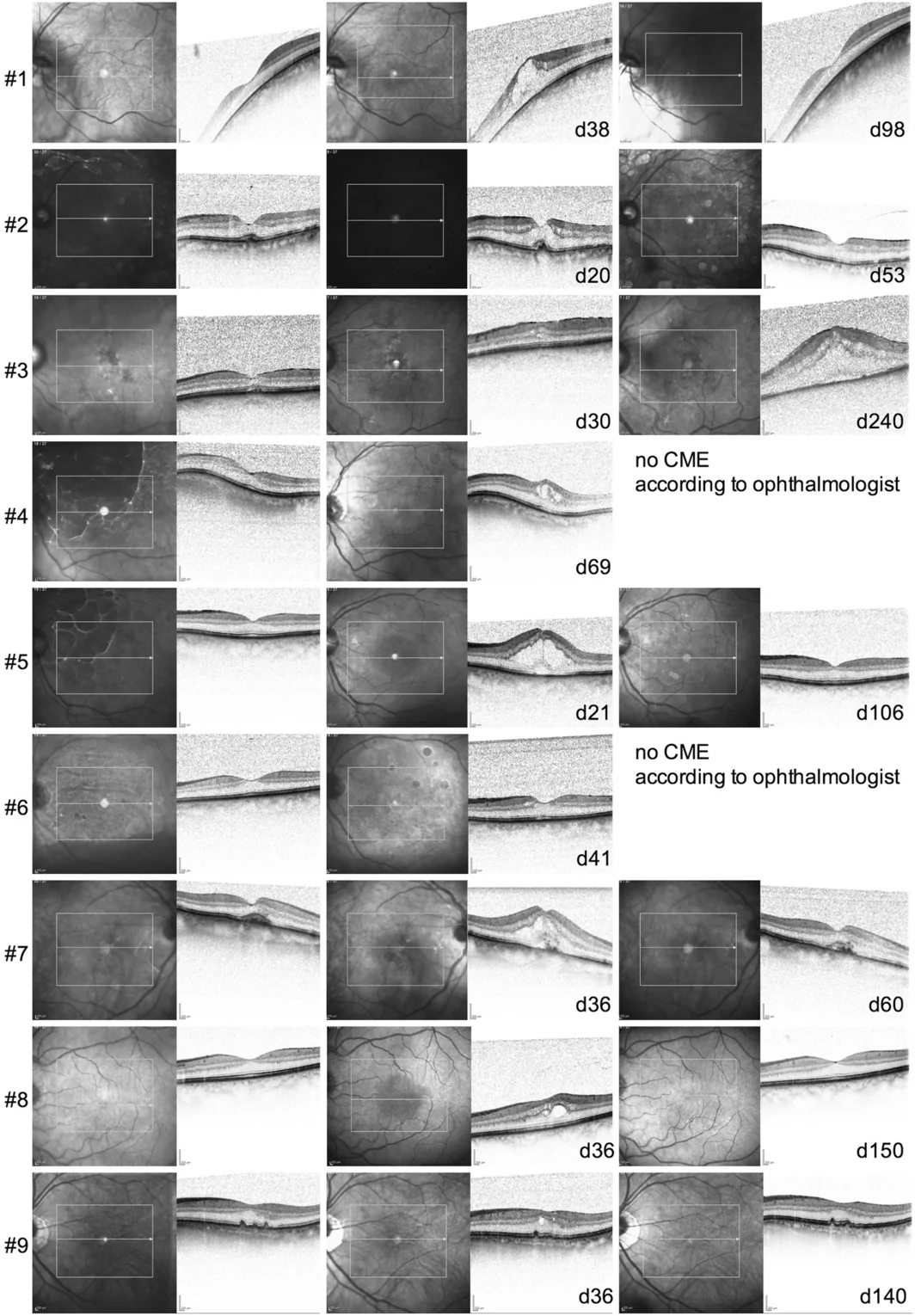
Cystoid macular edema (CME) after DMEK. Spectral‐domain optical coherence tomography images (OCT) of patients with CME after DMEK combined with cataract surgery (triple‐DMEK). Rows represent different cases; columns represent the OCT slice before surgery, at the first detection of CME and after treatment for CME, respectively. Lower right corner of each slice: Postoperative days (d). In 8 cases CME resolved under topical steroids. In 1 case (#3) CME persisted despite intravitreal steroids (dexamethasone 700 μg). In Cases #4 and #6 the patients' ophthalmologist performed the SD OCT. Development of CME after DMEK can be lowered significantly by early postoperative intense topical steroid therapy (Hoerster et al., 2016).

### The retrocorneal “fibrillar layer”

Which can be detected in the slit lamp by increased pigmentation and thickening of the stroma, is a significant risk factor for localized endothelial cell damage, oedema and glare (Hribek et al., 2021). The area can be detected in the Pentacam and correlates locally with significant collagen changes in the area of collagen 1, 3 and 4 (Hribek et al., 2021). The visualization of the fibrillar layer using Scheimpflug imaging may also allow for more personalized and even less invasive DMEK surgery in the future.

### DMEK in eyes with pre‐existing multifocal intraocular lenses (IOL)s

Gas or air instillation into the anterior chamber increases the risk of IOL calcification in hydrophilic acrylate lenses (Schrittenlocher et al., 2017). Nevertheless, DMEK can be performed in eyes with pre‐existing multifocal IOLs (Vishwakarma et al., 2025). However, visual acuity recovery is somewhat delayed, slightly reduced, and given the hydrophilic nature of most multifocal lenses, also associated with an increased risk of IOL calcification (Schrittenlocher et al., 2017). Alternatively, a temporary intracameral lens can be used to avoid gas‐lens contact with hydrophilic multifocal IOLs.

### What to do after graft failure in penetrating keratoplasty?

There are various treatment options:

- DMEK after penetrating graft failure is the gentler procedure, which also leads to faster visual acuity recovery. However, this option only makes sense if the visual acuity prior to graft failure was good without high astigmatism and there are no deep scars or vascular ingrowths. In a study of 52 DMEKs after graft failure in penetrating keratoplasty, we saw that the re‐bubbling rate is significantly higher than normal (59%) and that visual acuity recovery is worse than after “normal” DMEK. Preoperative OCT is essential for treatment planning. The risk of immune reaction is significantly increased to 13% after DMEK following endothelial failure of a pKPL (57; Schrittenlocher et al., 2020).
- DSAEK is similarly effective to DMEK, but there is an increased rate of graft failure.
- With re‐penetrating keratoplasty, the risk of rejection increases with the number of grafts.
- EndoArt can also be used in cases of transplant failure. In such cases, surprisingly high visual acuity recovery of up to 0.5 can sometimes be achieved.

### What are the more modern options for treating Fuchs' endothelial dystrophy?

Here, DSO (Descemet Stripping Only) is a treatment option in which the central Descemet membrane is removed in an area of 4–5 mm and then spontaneous migration of presumed healthy peripheral endothelial cells is awaited. The advantage of this is that there is no need for donor tissue. The disadvantage is the narrow indication and the delayed recovery of visual acuity, which is not entirely predictable.

Cell injections are a very interesting future therapeutic approach, but they are not yet widely available and appear difficult to implement due to their cost (Kinoshita et al., 2025).

Thank you very much for your attention and thanks to everyone at the Dept. of Ophthalmology at University of Cologne who participated in the studies presented here. Thanks also to the German Research Foundation (DFG) for supporting the Cologne Collaborative Research Center/SFB 1607 “Towards immunomodulatory and anti(lymph)angiogenic therapies for age‐related blinding eye diseases” (https://www.sfb1607.de/en/), thanks to the SFB team of researchers and clinician–scientist in Cologne and to the team at Cologne University Eye Clinic.

Thanks also to the Danish Ophthalmological Society (DOS) and its president, Professor Anders Ivarsen, for the honour of giving the 2025 Bjerrum Lecture.
